# Novel Pathogenic Variant c.258A>C, p.(Glu86Asp) in the *TTR* Gene in a Bulgarian Patient with Hereditary Transthyretin Amyloidosis

**DOI:** 10.3390/genes16070726

**Published:** 2025-06-22

**Authors:** Zornitsa Pavlova, Sashka Zhelyazkova, Mariana Gospodinova, Anastasia Ormandjieva, Tihomir Todorov, Ognian Asenov, Teodora Chamova, Plamen Antimov, Dilyana Mikova, Yordan Palashev, Ivailo Tournev, Albena Todorova

**Affiliations:** 1 Independent Medico-Diagnostic Laboratory Genome Center “Bulgaria”, 1612 Sofia, Bulgaria; 2 Genetic Medico-Diagnostic Laboratory Genica, 1463 Sofia, Bulgaria; 3 Department of Neurology, Expert Centre for Hereditary Neurologic and Metabolic Disorders, University Hospital “Alexandrovska”, Medical University of Sofia, 1431 Sofia, Bulgariateodoratch@abv.bg (T.C.);; 4 Expert Center for Transthyretin Cardiac Amyloidosis, University Hospital “St Ivan Rilski”, 1431 Sofia, Bulgaria; maryvg2009@yahoo.com; 5 Department of Nuclear Medicine, University Hospital St Ivan Rilski, 1431 Sofia, Bulgaria; 6 Clinical Center of Nuclear Medicine and Radiology, Medical University, 1784 Sofia, Bulgaria; 7 Department of Cognitive Science and Psychology, New Bulgarian University, 1618 Sofia, Bulgaria; 8 Department of Medical Chemistry and Biochemistry, Medical University Sofia, 1431 Sofia, Bulgaria

**Keywords:** ATTRv, *TTR* gene, pathogenic variant

## Abstract

Hereditary transthyretin amyloidosis (ATTRv) is an autosomal dominant disorder caused by pathogenic variants in the *TTR* gene. The destabilized mutant form of the transport protein transthyretin (TTR) leads to the extracellular deposition of amyloid fibrils. **Materials and Methods:** A 65-year-old female patient with suspected clinical diagnosis of ATTR was referred for genetic testing for pathogenic variants in the *TTR* gene after physical, neurological and cardiac testing. **Results:** The patient had had cardiac dysfunction, atrial fibrillation and supraventricular tachycardia for around 10 years before the suspected and confirmed cardiac amyloidosis. The molecular genetic testing showed a heterozygous pathogenic variant in exon 3 of the *TTR* gene NM_000371.4(TTR): c.258A>C, p.(Glu86Asp). This variant in the *TTR* gene is classified as pathogenic in accordance with ACMG/AMP for the interpretation of variants. **Conclusions:** The presented case of a very rare pathogenic variant in the *TTR* gene displays the valuable role of genetic testing on the way to clarifying a diagnosis.

## 1. Introduction

Amyloidosis is a group name for rare diseases with a common cause—the abnormal deposition of amyloid proteins, forming amyloid fibrils in various organs [1]. Hereditary transthyretin amyloidosis (ATTRv) is a rare subtype of the disease, in which the extracellular deposition of amyloid fibrils caused by the mutant form of transthyretin (TTR) produced in the liver affects mainly the heart, nerves and gastrointestinal tract [2]. ATTRv has autosomal-dominant inheritance and may be caused by more than 140 pathogenic variants in the *TTR* gene [3,4,5].

Age of onset and clinical phenotype are highly variable, as the disease symptoms can appear from the second decade of life until senility [6] and can range from sensorimotor and autonomic neuropathy to infiltrative cardiomyopathy [7].

In transthyretin amyloid cardiomyopathy (ATTR-CM), TTR builds up in the heart and affects the heart muscle, as well as the nerves and other organs [8]. The walls of the heart become stiff and the left ventricle is unable to relax and fill with blood. As a result, patients develop cardiomyopathy and heart failure [9]. Different variants in the *TTR* gene can cause differences in the progress of the disease and affect different organs. Because of this, genetic testing is a crucial step in the development of a treatment plan [7].

Timely diagnosis significantly improves the outcome of the disease, because a proper modifying therapy including TTR tetramer stabilizers [10] and gene silencing therapies (that reduce the mutant TTR production) [11] can be initiated.

ATTRv cases in Bulgaria are relatively high, with the most common *TTR* pathogenic variant among Bulgarian patients being NM_000371.3(TTR):c.325G>C, p.(Glu109Gln) [12,13].

Here, we present a clinical case of a 65-year-old female patient with a suspected clinical diagnosis of transthyretin amyloidosis, a carrier of a rare pathogenic variant in the coding sequence of exon 3 of the *TTR* gene.

## 2. Materials and Methods

**Figure 1:**
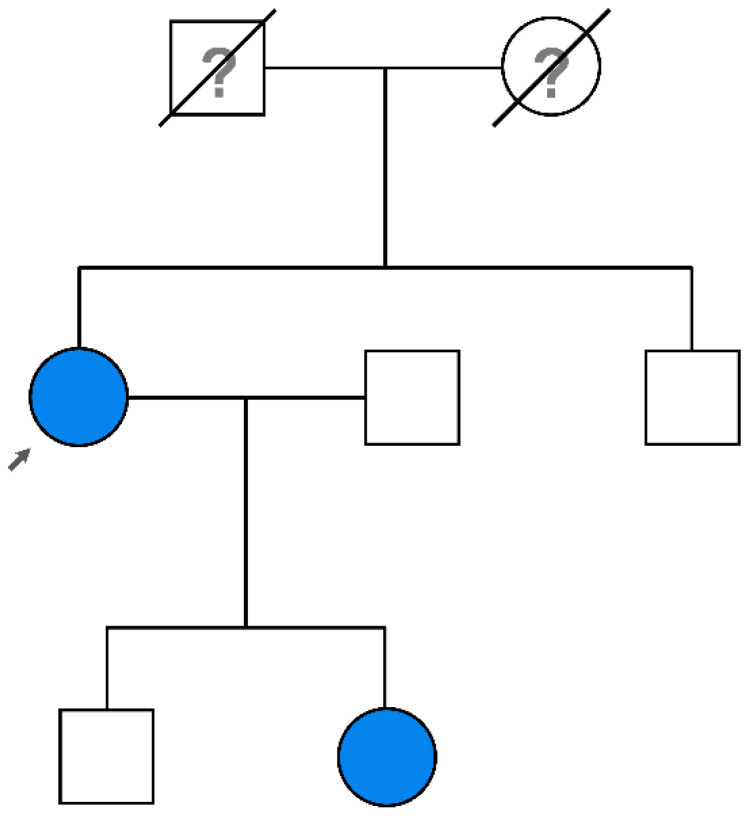
Heritage of the presented clinical case. The index patient is marked with an arrow; both the patient and her daughter are carriers of the *TTR* variant c.258A>C (the daughter was clinically asymptomatic at the time of genetic testing). The index patient’s parents are deceased from an unknown cause.

A proband patient (see Figure 1) underwent genetic testing after neurological and cardiological assessment suggesting ATTR diagnosis. Informed consent for genetic testing was obtained from the patient and her family.

The genetic testing involved Sanger sequencing of all protein coding regions of the *TTR* gene (NM_000371.4), which allows fast target testing of the patient. As long as all the known amyloidogenic pathogenic variants are located in the *TTR* coding sequence, this test is enough to confirm or exclude the hereditary form of ATTR.

Cardiac evaluation performed at baseline included 12-lead electrocardiography (ECG), transthoracic echocardiography, cardiac magnetic resonance and NT proBNP.

The patient underwent clinical evaluation at the time of diagnosis and a routine follow-up 6 months later, including neurological examination, Neuropathy Impairment Score (NIS) calculation [14,15], evaluation of familial amyloid polyneuropathy (FAP) stage and polyneuropathy disability score (PND) [16]. Neurophysiological tests included nerve conduction studies (NCS), sympathetic skin response (SSR) and electrochemical skin conductance (ESC), measured by Sudoscan [17]. Motor (tibial, peroneal, median and ulnar) and sensory (sural, superficial peroneal, median and ulnar) nerves were assessed bilaterally [18].

## 3. Results

The proband is a 65-year-old female, with clinical onset with tingling in both palms since the age of 49 years. She was diagnosed with bilateral carpal tunnel syndrome and subsequently underwent surgery on both hands 6 years later. At the same time, episodes of supraventricular tachycardia appeared with a frequency up to 190 beats/min and a treatment with Propafenone hydrochloride was initiated; this was subsequently changed to Sotalol hydrochloride. She also described early satiety and mild constipation. Since the age of 52 years, several attacks of atrial fibrillation had been recorded, and were treated with pharmacological cardioversion. At the age of 57, numbness and tingling in the feet appeared that were interpreted as radicular complaints related to disk herniations. At the age of 58 years, she started having frequent episodes of dry cough, especially in the evenings, with good response to corticosteroids. She was diagnosed with nonallergic asthma and started treatment with Montelukast per orally and inhaled corticosteroids. The patient also had a history of autoimmune thyroiditis with normal hormone levels, obesity, cholelithiasis and degenerative disk disease. The family history revealed that her father had pain, paresthesia and decreased sensation in the legs and died at the age of 79 years due to undetermined kidney disease. At the age of 65 years, the patient visited a cardiologist for routine cardiac assessment, but also complaining of progressive fatigue and dyspnea. A suspicion of cardiac amyloidosis was raised from the echocardiographic examination and she was referred for cardiac magnetic resonance imaging. CMR revealed left ventricular hypertrophy, extensive areas of late gadolinium enhancement involving all segments of the left ventricle, significantly increased T1 relaxation times and extracellular volume and relative preservation of the apical segments. Late gadolinium enhancement also affected the left atrium and right ventricle. These CMR findings suggested cardiac amyloidosis. The patient was referred to an expert center, where further evaluation by a multidisciplinary team was performed.

### 3.1. Physical Examination

On initial physical examination, the patient had blood pressure of 110/59 mmHg, a heart rate of 68 beats/min with regular rhythm, no audible murmur and rales on auscultation. There was no peripheral edema. The blood tests (complete blood count, liver enzymes, urea, blood glucose) and urine sediment test yielded normal results, except for serum creatinine level, which was slightly elevated—113 µmol/L (normal range 44–80 µmol/L). The abdominal ultrasound exam showed normal kidney sonographic appearance and no proteinuria was present.

### 3.2. Cardiac Evaluation

**Figure 2:**
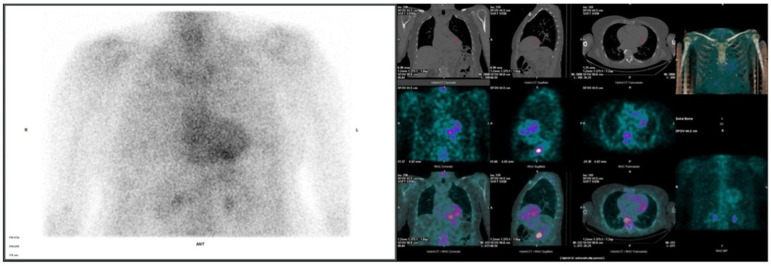
The 99mTc-pyrophosphate (99mTc-PYP) bone scintigraphy with SPECT/CT images; 99mTc-PYP myocardial uptake assessed as Grade 3.

The echocardiographic assessment showed significantly increased left ventricular wall thickness (≈19 mm), preserved ejection fraction (60%) and global longitudinal strain (−14.4%), stage II diastolic dysfunction, thickened valve leaflets, dilated atria, moderate mitral regurgitation, mild tricuspid regurgitation and no signs of pulmonary hypertension. The ECG revealed a sinus rhythm with a left axis and a discordance between the ECG voltage and left ventricular thickness. Following this, a 99mTc-PYP bone scintigraphy with a SPECT/CT scan was performed, revealing Grade 3 bone tracer uptake in the myocardium, with an N/CL ratio of 1.92 (Figure 2). Since the diagnosis of transthyretin cardiac amyloidosis was highly likely, the next crucial step was to rule out AL amyloidosis. The free light chain kappa and lambda values, as well as their ratio, were within the reference range, and no monoclonal protein was detected on immunofixation in the serum or urine. NT-proBNP was elevated at 1630 pg/mL, and GFR was 62 mL/min/1.72 m^2^. Following these tests, the diagnosis of ATTR amyloidosis with cardiomyopathy was determined. The patient was in NYHA class II-III, NAC Stage I. A genetic test was performed to differentiate between variant and wild-type disease.

### 3.3. Neurological Evaluation

Mild thenar muscle atrophy was present bilaterally, and was more pronounced in the left side. Plantar and brachioradial reflexes were attenuated. Pain and temperature sensations were decreased bilaterally in the distal parts of the four limbs. Vibration sense was absent in the feet and decreased in the legs and both hands. The position sense was decreased in the feet and preserved in the upper extremities. The neuropathy impairment score total was 18 points. NCS was consistent with axonal sensory and motor polyneuropathy in the four limbs and bilateral carpal tunnel syndrome. The SNAP from the superficial peroneal nerves were unobtainable, while for the median nerves they were decreased. The amplitude of CMAP was decreased for both peroneal nerves as well as for the median nerves. The distal latencies of SNAP and CMAP of the median nerves were prolonged. Sudoscan was normal. A sympathetic skin response test showed no responses after electric stimulation in the right hand and both feet.

### 3.4. Genetic Testing

Molecular genetic analysis revealed the presence of a heterozygous pathogenic variant in exon 3 of the *TTR* gene NM_000371.4(TTR): c.258A>C, p.(Glu86Asp). This variant is not detected in the gnomAD control population and is not reported in the ClinVar database. The c.258A>C, p.(Glu86Asp) variant in the *TTR* gene is classified as pathogenic according to the ACMG/AMP variant interpretation guidelines (categories: PS1, PM1, PM5, PM2 and PP3) [19]. The genetic test confirmed the diagnosis of Transthyretin Amyloid Cardiomyopathy (ATTR-CM) with NM_000371.4(TTR): c.258A>C, p.(Glu86Asp) as the causative pathogenic variant.

### 3.5. Treatment

Treatment with Tafamidis was initiated. The patient was treated with a loop diuretic, aldosterone antagonist and an SGLT2 inhibitor. We have been following the patient for 6 months, with no signs of heart failure exacerbations. The neurological involvement remained stable and the serum creatinine level slightly decreased (from 113 µmol/L to 105 µmol/L).

## 4. Discussion

We present a clinical case of a Bulgarian 65-year-old female patient diagnosed with ATTR-CM, with a significantly rare variant in exon 3 of the *TTR* gene, c.258A>C, p.(Glu86Asp). A similar variant has been reported only once in the literature as a novel variant in a female Chinese patient. Cheng et al. reported ATTR-E66D variant (c.258A>T, p.(Glu86Asp)) as a novel variant in a patient from their multiracial South-East Asian cohort from Singapore [20]. We report a nucleotide change from adenine to cytosine (c.258A>C), but unlike us, Cheng et al. reported a change from adenine to thymine (c.258A>T) [20]. However, these changes lead to the same amino acid substitution p.(Glu86Asp).

In our case, the age at onset of the disease was earlier than in the Chinese patient (49 years compared to 69 years), with initial involvement of the upper limbs, with bilateral carpal syndrome, followed by the lower ones. The neurologic symptoms were mild; mainly sensory symptoms with preserved muscle strength. Regarding gastrointestinal symptoms, the Chinese patient was reported to have diarrhea and gastroparesis while our patient had only early satiety and mild constipation. Cheng et al. reported that the patient had no positive family history [20]. However, in the current case, there is a positive family history for sensory disturbances in the legs and renal failure from the patient’s father. The renal involvement in our patient consisted of slightly increased serum creatinine with no proteinuria. The data on kidney involvement in ATTRv is highly variable in the literature, as nephropathy was described in one third of the patients from a large Portuguese cohort with Val30Met mutation [21]. Solignac et al. suggests that the Val122Ile mutation could be an important risk factor for chronic kidney disease [22]. Further studies are needed to determine the role of the pathogenic variant c.258A>C, p.(Glu86Asp) or the development of nephropathy.

Genetic testing of the family revealed that the daughter of the proband (Figure 1) inherited the pathogenic variant, and at the time of testing she was asymptomatic. As for cardiac dysfunction, our patient had atrial fibrillation and supraventricular tachycardia around 10 years before the suspected cardiac amyloidosis and final diagnosis. The Chinese patient had congestive cardiac failure at 72 years old and a sural nerve biopsy identified transthyretin amyloid deposition in epineural blood vessels and connective tissue [20].

## 5. Conclusions

Here, we present a novel pathogenic variant in the *TTR* gene in a patient diagnosed with ATTR-CM. A similar *TTR* variant affecting the same nucleotide position has been reported only once as a novel variant [20]. The presented case report highlights the necessity of local and global collaboration for genotyping ATTRv in order to find other possibly unknown *TTR* variants that can be classified as pathogenic and can have an impact on the treatment plan and be added in new treatment clinical trials.

## Data Availability

The raw data supporting the conclusions of this article will be made available by the authors, without undue reservation.
